# Enhancing Performance in Young Athletes: A Systematic Review of Acute Supplementation Effects

**DOI:** 10.3390/nu16244304

**Published:** 2024-12-13

**Authors:** Nina Gruska, Hugo Sarmento, Diogo Martinho, Adam Field, Alain Massart

**Affiliations:** 1 Research Unit for Sport and Physical Activity, Faculty of Sport Sciences and Physical Education, University of Coimbra, 3040-248 Coimbra, Portugal; hugo.sarmento@uc.pt (H.S.); dvmartinho92@hotmail.com (D.M.); alainmassart@fcdef.uc.pt (A.M.); 2 Department of Sport and Exercise Science, Institute of Sport, Manchester Metropolitan University, Manchester M1 5GD, UK; a.field@mmu.ac.uk

**Keywords:** pre-training supplementation, acute effects, young athletes, ergogenic substances, teen, children, carbohydrate, beetroot juice, sodium citrate

## Abstract

Background/Objectives: The increasing popularity of acute supplementation among young athletes is concerning, given the limited scientific evidence to guide recommendations specific to this group. Therefore, the aim of this systematic review was to synthesize the available scientific evidence on the acute effects of supplementation in young athletes to understand the impact on physical and cognitive performance. Methods: Following pre-registration on INPLASY (INPLASY202310017) and according to the PRISMA (Preferred Reporting Items for Systematic Reviews and Meta-Analyses) guidelines, systematic searches of three electronic databases (Web of Science, PubMed, and Scopus) were conducted by independent researchers from inception until July 2024. Only original studies in English that examined the acute effects of supplementation on young athletes’ physical and/or cognitive performance, specifically when taken on the same day as exercise (before or during), were included. The risk of bias was individually assessed for each study using the revised Cochrane risk of bias tool for randomized trials (RoB 2.0). Results: A total of 16 studies were included in the review. A range of ages, participants, sports, and methodological approaches were involved in the studies included in the current review. The studies mainly used carbohydrates, beetroot juice, and sodium citrate, with carbohydrates being the most used acute supplementation. Carbohydrate supplementation enhanced endurance capacity and increased blood glucose, but mixed results were found for anaerobic performance. Mixed results were found for beetroot juice, with one study finding increases in power production. One study used sodium citrate supplementation, with improvements in technical performance observed. Conclusions: Since this review identified only three substances meeting our eligibility criteria, further research is needed to confirm the acute effects of supplements in young athletes and to better understand their benefits and limitations. Carbohydrate supplementation shows strong evidence for enhancing endurance performance, particularly during prolonged activities, while sodium citrate appears to support the preservation of skill performance. In contrast, the effects of beetroot juice are less consistent. Additional research is required to confirm the acute effects of supplements like beetroot juice in young athletes.

## 1. Introduction

Ergogenic aids are substances intended to enhance sports performance and provide additional benefits, such as supporting muscle recovery, preventing injuries by promoting joint and muscle health, and assisting in managing training loads to optimize physical adaptations [1,2]. These supplements might not only enhance performance during competition but may also play a crucial role in maintaining overall athlete health and longevity by potentially reducing the risk of injury and supporting recovery processes. Athletes consume these supplements to enhance training adaptations, immune functioning, recovery, cognition, and decrease perceptions of fatigue [3,4]. Although supplements such as carbohydrates, protein, creatine, caffeine, beta-alanine, sodium bicarbonate, tart cherry, and beetroot juice are commonly sought after by athletes for enhancing sports performance, not all of these supplements have benefits that are fully evidence-informed [3]. Therefore, it is important to investigate the acute effects of ergogenic supplementation before implementing them in practice [5,6]. Various factors should be considered before the prescription of supplements to improve performance, including reducing the risk of doping infringements and health damage due to ingesting contaminated ingredients [7].

Young athletes undergo various stages of growth and maturation [8,9]. Therefore, applying the same supplementation guidelines designed for adult athletes could potentially influence the progression of performance in young athletes by altering the natural developmental trajectory or introducing risks associated with inappropriate use [9,10,11]. These effects may arise due to differing nutritional requirements compared to adults, which can be either higher or lower, or from the potential for excessive consumption relative to their specific nutrient needs [12,13]. Especially since it is thought that young athletes mirror the attitudes of those more experienced and adopt similar supplementation protocols that are recommended for adults [9,14]. Young athletes who excel in their fields deserve special attention due to the strenuous energy expenditure from intense physical training and the energy demands for growth and development [10,15]. One of the main objectives of coaches and parents of young athletes is to understand whether the training demand and nutritional intake are optimal for physical development, performance, and recovery. However, it is crucial for athletes to meet the nutritional demands of their sport, and we cannot overlook the impact of supplementation on sports performance [16].

Given the challenges in providing appropriate supplementation for young athletes, there is limited scientific evidence to develop supplementation guidelines for this population [9,17]. To fulfill, in part, this significant gap, the aim of our systematic review is to identify the studies published on acute (on the same day and/or before/during exercise) supplementation impacts on physical and/or cognitive performance in young athletes. Through this review, we hope to propose effective acute supplementation strategies that support young athletes in enhancing performance.

## 2. Materials and Methods

### 2.1. Protocol and Registration

The current review was conducted according to PRISMA (Preferred Reporting Items for Systematic Reviews and Meta-Analyses) guidelines [18]. The methods were established before protocol registration and conducting the searches. The protocol was registered on INPLASY^®^ (registration number: INPLASY202310017).

### 2.2. Eligibility Criteria

Studies were eligible if they constituted original research or replication studies published in peer-reviewed journals, with no limitations on language or publication date. The eligibility criteria used in this review were: (1) studies supplementing acutely (i.e., on the same day, before or during); (2) studies involving either competitive or recreational athletes under 18 years; (3) studies that included male and/or female participants competing in any sport; and (4) studies that assessed the effects of supplementation on physical and/or cognitive performance. The exclusion criteria included: (1) studies that did not report the athletes’ age; (2) review articles; (3) unpublished studies in scientific journals (e.g., abstracts, dissertations, or theses); (4) studies that included the use of supplementation after performance measures; (5) studies including individuals with medical conditions; (6) articles that did not report the dosages of the supplementation; (7) studies including substances that could affect young athletes’ health (i.e., studies involving caffeine and/or energy drinks were excluded due to evidence of potential adverse effects, such as impaired sleep latency and hormonal imbalances, which could negatively impact young athletes).

### 2.3. Information Sources and Search Strategy

The search was carried out on 9 July 2024. The results were exported to EndNote version 20.6 for Windows X9 (Clarivate Analytics, Philadelphia, PA, USA). The electronic databases searched were Web of Science (all databases), PubMed, and Scopus. The following terms were used: (Sport* OR exercise* OR athletic OR soccer OR swim* OR tennis OR gymnastic* OR judo OR basketball OR rugby OR football OR “team sport”) AND (“ergogenic effect” OR “ergogenic aid” OR “ergogenic substance$” OR “dietary supplement$” OR “food supplement*” OR carbo* OR resveratrol OR taurine OR beetroot OR ATP OR phosphocreatine OR choline OR magnesium OR vitamin*) AND (Performance) AND (youth OR young OR kid* OR child* OR “pre puberty” OR “young athlete”). A manual search was performed on the reference lists of the records included to retrieve potentially relevant studies that were not located in the initial searches. After the list of studies was finalized, all databases were revisited to retrieve errata, corrections, or retractions of the included studies.

### 2.4. Selection Process

NG and HS conducted the initial screening process, which included title and abstract screening and full-text analyses. The selection process included: (1) identification of duplicate articles; (2) title analyses; (3) abstract analyses; (4) full reading of the articles considering the inclusion and exclusion criteria. Disagreements between researchers were solved by consensus through the third investigator (AM).

### 2.5. Data Extraction Process

All extracted data were defined a priori to avoid biased analyses. Study characteristics included: (1) sample size and characteristics (i.e., age, sex, health, sport/modality, type of exercise, location); (2) supplementation (i.e., vitamins, minerals, any ergogenic supplement); (3) protocol (i.e., acute use, dosage, objective(s), study procedures, methodology, experimental design, placebo used, collection instruments, feeding, hydration, measured outcome, main result); (4) frequency (i.e., daily consumption, minutes before competition(s) or event(s)); (5) objective of supplement (i.e., improvement in physical/mental performance, inflammatory markers, blood glucose, perception of fatigue). The primary outcomes of the review article relate to physical performance (e.g., maximum repetitions, time to exhaustion, distance traveled, VO2max, strength, power, and speed). The secondary outcomes included mental and physiological fatigue (e.g., fatigue perception, concentration, inflammatory markers, sleep-related factors, and muscle recovery). The effects of supplementation on subjective measures were also considered as tertiary measures (e.g., palatability of the supplementation, comfort or discomfort ratings, stress, and eating habits).

### 2.6. Quality Assessment and Risk of Bias

The revised Cochrane risk of bias tool for randomized trials (RoB 2.0) was used to analyze the risk of bias in the randomized studies included in the current review. This tool covers five domains, each with different questions: (1) bias arising from the randomization process; (2) bias due to deviations from the intended interventions; (3) bias due to missing outcome data; (4) bias in the measurement of the outcome; (5) bias in the selection of the reported result. In this review, we used the tool to assess the impact of the initial interventions and adherence to them, as outlined in the protocols of the trials analyzed. For each question of the tool, we assigned one of the following responses: yes, probably yes, probably no, no, or no information. Each domain was categorized as having a low risk of bias, some concerns, or a high risk of bias. Each domain was completed wherever the information was available. The assessment of the tool was conducted by two independent observers (NG/DM), with any disagreements being resolved by a third and fourth reviewer (AM/HS).

## 3. Results

### 3.1. Study Selection

**Figure 1:**
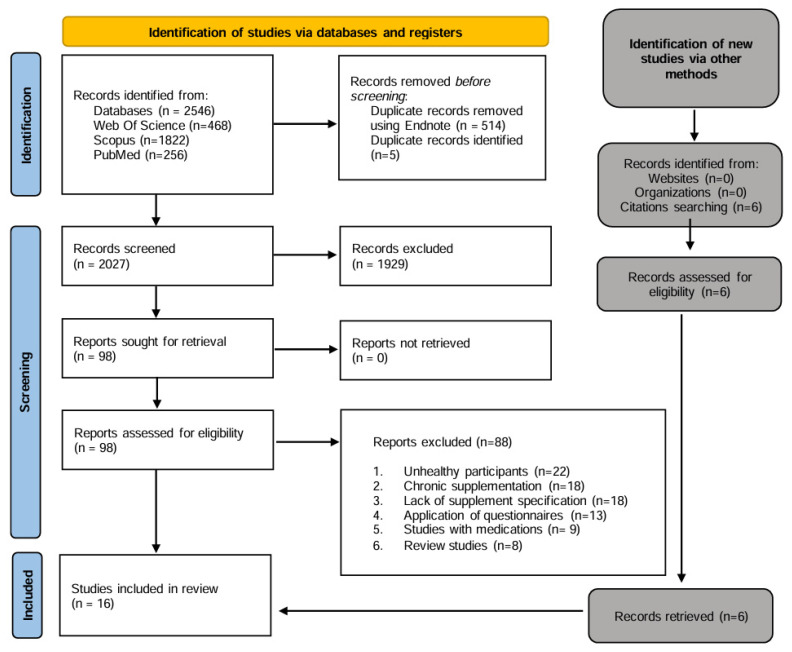
PRISMA flow diagram.

The initial searches resulted in a total of 2546 studies (Web of Science—468, Scopus—1822, PubMed—256). This process, along with the keywords and filters applied, is detailed in Supplementary Table S1. The identification of duplicates was initially conducted automatically using EndNote, which identified 514 duplicates. Subsequently, the first and last authors (NG/AM) manually identified an additional 5 duplicates, resulting in the removal of a total of 519 duplicates. This process reduced the total number of articles for screening to 2027. The first stage of title and abstract screening was based on the type of study and resulted in the exclusion of 1929 records. A total of 98 studies were read in full, with 88 studies excluded for not meeting the eligibility criteria. A further six articles met the eligibility criteria following manual searches. In total, 16 articles met the eligibility criteria and were included in the review. The process is summarized in the PRISMA flow diagram (Figure 1).

### 3.2. Study Characteristics

The sample size varied across studies from seven to fifty-eight participants [11,19]. The participants’ ages ranged from 10 to 17 years [10,16,17,18,19,20,21,22,23,24,25,26,27,28,29,30,31,32]. Three studies conducted their analyses with both boys and girls [11,19,20]. Heidorn et al. [21] and Timmons et al. [12] conducted tests solely on girls. All the other studies were conducted only in boys.

Six studies were conducted in Europe [11,20,22,23,24,25], eight studies in North America [13,21,26,27,28,29,30,31], one study in South America [32], and one study in Asia [19].

Several studies conducted interventions in competitive athletes [11,20,22,23,24,25,28,32], while six articles involved individuals described as ‘active’ [12,13,19,21,26,31]. Bender et al. [27] did not specify the competitive level of the adolescent participants. Marjerrison et al. [29] evaluated a set of active individuals in which some participants were defined as being ‘competitive’ athletes.

Heidorn et al. [21] and Guth et al. [26] included a controlled intervention. Four articles implemented a randomized crossover analysis [19,23,25,29]. The other studies implemented a double-blind experimental design [11,13,20,22,24,27,30,31,32].

Thirteen articles assessed the impact of carbohydrates on the performance of young athletes. The mode of carbohydrate administration varied across these studies [11,13,19,20,21,23,24,25,26,28,29,30,31]. Phillips et al. [20] provided carbohydrate in gel form. One study used dextrose [26], six studies provided sucrose [13,19,21,23,30,31], and four studies administered maltodextrin [11,20,21,24]. Lee et al. [28] and Marjerrison et al. [29] used commercial beverages with glucose, sugar and other additives. Two studies examined the effects of beetroot juice on the performance of 16-year-olds [22,27]. Only one study analyzed the effects of sodium citrate on the performance of 17-year-old tennis players [32].

Phillips et al. [24] provided 0.8 g·kg^−^^1^ BM of carbohydrates, whereas Marjerrison et al. [29] utilized 1.0 g·kg^−^^1^ BM. Comparatively, Timmons and Bar-Or [30] administered 1.4 g·kg^−^^1^ BM, while subsequent investigations by Timmons et al. [13,31] employed 1.7 g·kg^−1^ BM of carbohydrate, respectively. Lee et al. [28] used 1.5 g·kg^−1^ BM of carbohydrate, and Russell et al. [23] provided a total of 30 g of carbohydrate-electrolyte with a standardized meal. Heidorn et al. [21] supplied approximately 19 g of carbohydrate. Stough et al. [19] used a combination of 11 g malt extract, 6.5 g milk, and 0.44 g sucralose sweetener. Guth et al. [26] administered a solution with 6% carbohydrate. Phillips et al. [20] used 0.8 g·kg^−1^ BM carbohydrate, while Carvalho et al. [25] allowed ad libitum intake of an 8% carbohydrate-electrolyte solution.

In the context of beetroot juice, both Bender et al. [27] and Lopez-Samanes et al. [22] provided participants with 140 mL, which was standardized to contain 400 mg of nitrate. Lastly, Cunha et al. [32] investigated sodium citrate, using 0.5 g·kg^−1^ BM of carbohydrate.

Lopez-Samanes et al. [22] and Carvalho et al. [25] conducted research in youth basketball athletes. Russell et al. [23] analyzed football players. Cunha et al. [32] incorporated young tennis players. Some research included ‘active’ teenagers but did not specify the sports in which the participants competed [13,21,26,27,29,30,31]. Phillips et al. [20], Phillips et al. [11], Phillips et al. [24], and Lee et al. [28] grouped athletes from team sports (football, rugby, basketball, and hockey). A significant variability was found in the supplementation timings in the included studies. Supplementation was provided 5 min [11,20,21,24] and 30 min before performance was measured [28,29].

Carbohydrate supplementation demonstrated positive effects on performance in six studies [11,13,20,24,28,31]. The characteristics of the studies included in the present review are summarized in Table 1. The protocols and results of the studies included are summarized in Table 2.

### 3.3. Risk of Bias

**Figure 2:**
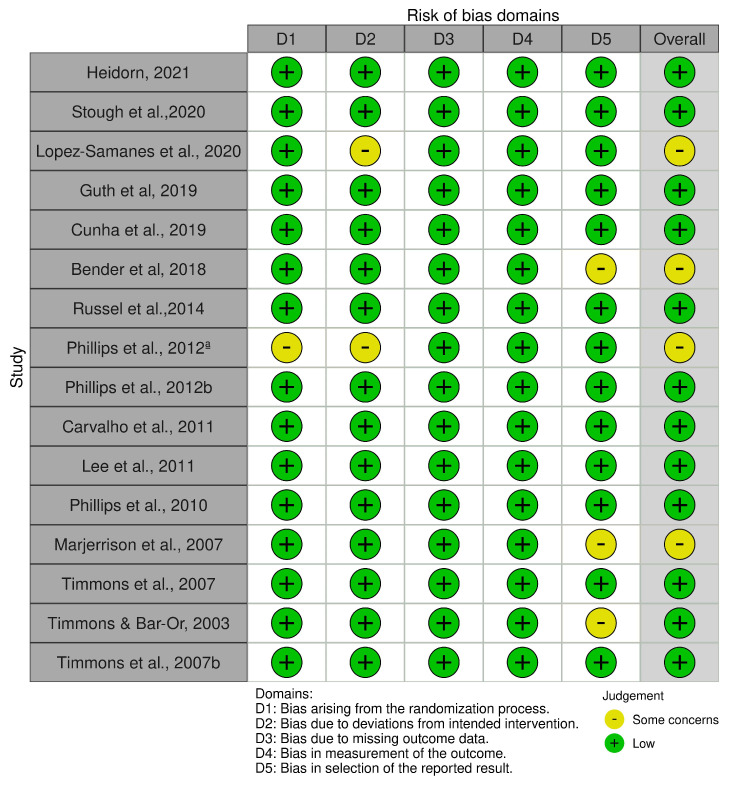
Risk of bias for each study using the revised Cochrane risk of bias tool for randomized trials. D1: Bias arising from the randomization process, D2: Bias due to deviations from intended interventions, D3: Bias due to missing outcome data, D4: Bias in measurement of the outcome, D5: Bias in selection of reported results. CHO: Carbohydrate; CHO-E: Carbohydrates with Electrolytes; HCHO: High Carbohydrate; LCHO: Low Carbohydrate; VIE: Variable Intensity Exercise [11,13,19,20,21,22,23,24,25,26,27,28,29,30,31,32].

In general, the studies demonstrated a low risk of bias across all five domains. However, the details of the randomization process were not adequately clarified in one study [17]. None of the included studies showed significant limitations related to bias due to deviations from the intended interventions, indicating a low risk of bias in this domain. Some concerns were evident in one study [17] regarding bias in measuring the outcome due to the small number of participants. However, the article indicated that the observers were not aware of the treatment and control groups. Concerns about bias in the selection of reported results were identified in three studies [19,28,30]. Not all outcomes were included (i.e., speed, fatigue, heart rate, perceptions of effort). One study [16] reported improvements in cognition. Only key findings were integrated into the analysis (Figure 2).

## 4. Discussion

This systematic review aimed to investigate the acute effects of supplementation on performance in young athletes. The studies included in the review included heterogeneous participants with varied maturational levels, sexes, sample sizes, sports, and methodological approaches. The review encompasses studies with diverse methodologies, including varying exercise intensities, testing environments, and supplementation protocols. While this diversity reflects real-world scenarios, it also complicates direct comparisons and synthesis. The studies included in the review used carbohydrates, beetroot juice, and sodium citrate.

Carbohydrate supplementation consistently improved endurance capacity and increased blood glucose, but mixed effects were found for anaerobic performance. Equivocal findings were reported for beetroot juice, with a single study finding that power production was increased. The initial physical condition may influence the effectiveness of beetroot juice on anaerobic performance, with experienced athletes showing a limited response and recreationally active adolescents demonstrating greater strength gains, possibly due to lower prior physical adaptation [22,27]. The study assessing sodium citrate supplementation found improvements in shooting consistency [32].

Although our review established an age criterion for young individuals, it is essential to consider the complexities associated with maturation and development within this age group. A significant gap in the literature was found during the current review, which precluded adequate filtering of samples by age. Factors such as physical development and maturation can vary in young people. These factors can also affect growth, metabolic, and physiological capacities, and consequently, can influence supplementation outcomes. Notably, the impact of acute supplementation in girls was analyzed in only two studies [13,21]. One of these studies compared girls and adolescent females, demonstrating that exogenous carbohydrates improve glycogen sparing and reduce post-exercise lactate, highlighting potential benefits for prolonged efforts across maturational stages and sexes [13].

In boys, Timmons et al. [31] investigated how chronological age and pubertal stage influence substrate utilization during exercise, emphasizing that pubertal maturation has a greater impact than age. Prepubertal and early pubertal boys demonstrated a higher reliance on exogenous carbohydrates and better glycogen conservation, whereas mid- to late-pubertal boys exhibited increased endogenous carbohydrate oxidation. Additionally, Guth et al. [26] observed that young boys responded differently from adults, with boys who received the carbohydrate drink showing increased glycemia at 24 and 36 min of exercise.

Building upon previous findings, the study by Phillips et al. [11] has notable limitations, including considerable variability in participant body weight (62.0 ± 6.3 kg) and a small sample size (n = 7). The weight discrepancy likely influenced the carbohydrate dose consumed, as the beverage, containing 6% carbohydrate, was administered based on body mass (mL/kg), potentially introducing variability in the outcomes. Additionally, the small sample size reduces statistical power, and these factors call into question the conclusion that the 6% carbohydrate drink is the most effective.

In this review article, considerable methodological differences were reported across the studies. For instance, a wide range of exercise intensities was used in two studies [21,26], while a fixed-duration Wingate protocol was used in three studies [27,28,29]. A range of performance outcomes were used, including, the countermovement jump [22], a T-test [22], a simulated soccer match (termed the LIST) [11,20,24], and sport-specific movements and motor skill assessments [22,25]. Stough et al. [19] evaluated a group of younger children and applied playful activities involving badminton, squash, football, and a “hopping relay” skill. Despite the larger sample size compared to other studies analyzed, the evaluation methodology of Stough et al. [19] was the most complete for incorporating playfulness, adapting movements to the age group, and analyzing impacts on cognitive capacity. These types of assessments enhance testing reliability since the athletes are familiarized with the movements. This methodological approach to acquainting participants with the testing procedures was not apparent and/or reported throughout the studies. As such, learning effects may have occurred during some of the protocols used.

Two double-blind studies involved the application of various micronutrients and examined the performance of both children and young people [11,24]. In the study by Phillips et al. [24], both groups received electrolytes, containing 250 mg sodium, 60 mg magnesium, 90 mg potassium, and 20 mg calcium. Although food and carbohydrate intake were not controlled the day before and the day of the tests, participants were advised to have the same meals with similar portions on both days. This tends to be a method of dietary standardization used across the studies included in this review, particularly in experimental trials involving crossover designs with a placebo and intervention group. It is crucial to ensure that there are no differences in carbohydrate and energy intake between the intervention and placebo groups in the 24-h period preceding both trials. Most studies lacked dietary control [11,19,20,21,24,25,26,27,29,32], which may confound the results, as the benefits of carbohydrate intake on performance are well-established [4,33,34].

Carbohydrate intake’s effect on intermittent endurance and sprint performance was assessed, with participants consuming either 0.818 g/kg BM of carbohydrate gel or a placebo [20]. While sprint times and heart rate showed no significant changes, carbohydrate intake notably improved intermittent endurance capacity, allowing participants to sustain performance for a longer duration during a simulated team game. Carbohydrate gels are better accepted among athletes due to their practicality and digestibility [35]. One of the possible benefits of carbohydrate gels is their ability to offer a large amount of carbohydrates in smaller volumes [1]. Even though the intervention carried out by Phillips et al. [20] did not influence average sprint time, more studies could utilize carbohydrate gels due to their ability to offer high concentrations of carbohydrates in smaller portions. This makes it easier to administer the carbohydrates during competition intervals.

Mettler et al. [36] and Desbrow et al. [17] recommend the requirements for a young athlete during a growth phase while engaging in strenuous physical exertion. These recommendations suggest that children and adolescents require high energy and micronutrient demands to provide energy during peak periods of growth and development [37]. When they are exposed to training with a high energy demand and experience stress associated with competitive environments, it is plausible to assume that carefully considered supplementation would appear warranted to provide additional benefits. When analyzing studies with questionnaires [38,39,40,41], the intake of supplements is part of the routine of many young athletes, especially those at a competitive level. For instance, a recent study conducted in Switzerland involving nearly 500 elite adolescent athletes indicated widespread and large-scale use of dietary supplements, with the most commonly used being vitamin supplements, carbohydrate and electrolyte drinks, creatine, protein bars, and energy drinks. The study also concluded that the population had a low level of knowledge about the effects and purposes of supplementation [36]. Evidence also suggests that the most influential factors of supplementation are purported to be parents and coaches [42], and a large proportion of this population is unaware of the adverse effects of supplementation and unsure of the appropriate amounts and supplements suitable for young athletes [43].

It is challenging to determine the impact that a single acute supplementation may have on young individuals’ health outcomes [44]. For instance, the caffeine content in energy drinks often exceeds the recommended quantities for children and adolescents, which can impair sleep latency and, consequently, muscle growth, mental rest, and hormone production essential for this stage of life. Although caffeine was not addressed in this review, single-use instances in younger individuals can be harmful due to their smaller body mass and sensitivity to dosage [45]. Understanding the short-term effects of these supplements, especially during competitions or intense training, is also important. Even small, acute improvements in performance may, over time, support meaningful adaptations and might enhance overall athletic development [46]. While there are a wealth of studies involving the use of supplements, the efficacy of these substances remains equivocal, as there is a lack of research in young athletes. If these supplements are natural and safe for consumption—such as beetroot juice and other whole foods—meaning they are composed of non-toxic ingredients, free from preservatives, added ingredients, artificial colorings, and sugars, and are formulated with appropriate dosages for young athletes, then more research should be undertaken on their use in this population [47].

Adolescents and children have distinct energy and supplementation needs compared to adults, due to their unique growth and maturation characteristics [48]. These physiological variations become even more evident when examining energy and macronutrient intake in young athletes, which must be adjusted to meet specific growth and developmental needs, taking into account both maturation stage and training volume [49,50,51].

The variability in performance progression among young athletes within the same chronological age category reflects significant differences in growth, maturation, and training response, and consequently in their responses to supplementation [31,52]. Relative age effects favor those born earlier, resulting in the selection of athletes with average or advanced biological maturity, who tend to exhibit greater muscular strength, motor performance, and VO2max compared to their later-maturing peers [49,53]. As individuals mature, physiological changes such as increased hormone production, muscle development, and improved metabolic efficiency directly influence how the body metabolizes nutrients and responds to supplementation [50,53].

These maturation differences not only affect supplementation responses but also how dosages adjusted by kg of body mass should be considered. Early-maturing boys tend to exhibit more pronounced responses to supplements like carbohydrates and proteins due to higher hormonal levels [48,51]. In the present systematic review, the samples included participants at different Tanner stages, with seven studies failing to report maturation status, highlighting important methodological limitations that hinder the interpretation of findings from supplementation studies involving young athletes [19,21,22,23,25,27,32].

Despite the challenges in this area of research, this review showed where focus could be directed in future studies. No prospective studies were eligible for inclusion in the current review (i.e., studies that follow a group of people over time). Nonetheless, these types of studies are crucial to determine the cause-and-effect relationships and to capture changes in behaviors over time and during specific periods of training like competition or post-competition.

## 5. Limitations

This systematic review presents some limitations that affect the practical applicability of acute-use supplements and highlight the need for further research focused on young athletes. First, the included studies used a variety of performance metrics, such as endurance capacity, power, sport-specific skills, and cognitive performance. While this diversity captures the complexity of athletic performance, it also complicates direct comparisons, weakening conclusions about the efficacy of specific supplements.

Additionally, although we used a variety of alternative keywords, we acknowledge that some relevant terms may have been overlooked, potentially limiting the scope of our search and excluding potentially important studies. We suggest that future reviews consider a more comprehensive set of terms to ensure broader literature coverage.

Furthermore, although the aim of the review was to evaluate both physical and cognitive performance, only one study actually explored cognitive outcomes. Given the importance of cognitive development in young athletes, a more detailed analysis of how supplements may influence cognitive functions during athletic activities would provide a more comprehensive understanding of supplementation effects. Another significant challenge is the small sample sizes in many of the studies analyzed, which may contribute to the variability observed in the results. Small samples represent a particularly notable limitation when evaluating complex performance outcomes, as individual variability can hinder robust conclusions. Additionally, inconsistencies in timing and dosage protocols for supplementation across studies add another layer of complexity. While some studies assessed effects immediately after ingestion, others introduced supplementation during exercise, which can affect absorption rates and peak efficacy, making it challenging to establish ideal timing and dosing for young athletes.

Finally, the limited use of advanced physiological assessments and reliance on subjective measures, such as perceived exertion, further complicates the interpretation of results. Objective metrics, such as VO2max, inflammatory markers, blood glucose monitoring, and GPS tracking, could provide more precise insights into the physiological effects of supplements but were rarely included. The lack of dietary control in many studies also represents a significant limitation, introducing the potential for confounding factors that interfere with the observed effects of supplementation.

## 6. Practical Applications

Although supplementation is often used to enhance performance, some studies included in this review reported side effects that should be considered when recommending such strategies for young athletes. For example, gastrointestinal discomfort was a commonly reported adverse effect, particularly with higher doses of sodium citrate [32]. Nausea and bloating were observed in a few participants consuming concentrated nitrate solutions, which may limit their practical application in competitive settings [22,27]. Additionally, transient increases in perceived exertion and heart rate were noted with carbohydrate supplementation during prolonged or intense activities, potentially indicating a mismatch between energy supply and utilization [23,26]. Manipulating the carbohydrate concentration of the ingested solution has been associated with an increased risk of gastrointestinal distress, as highlighted by Phillips et al. [11]. Although no severe adverse events were reported, these findings underscore the importance of individual tolerance and dose adjustments when using supplements to support the performance of young athletes.

Despite these considerations, carbohydrate supplementation is a well-established strategy to enhance endurance and maintain blood glucose levels during prolonged exercise, particularly in long-duration activities. Consuming 1 g/kg–1.5 g/kg of body mass (BM) approximately 30 min prior to exercise provides a reliable energy source and may contribute to improved performance, particularly in adolescents. However, it is crucial to use these strategies with caution, as individual responses can vary, and the absence of severe adverse effects in the studies reviewed does not guarantee safety across all contexts.

The ingestion of approximately 0.8 g/kg of carbohydrates immediately before exercise and in fractional amounts during exercise has been shown to be especially beneficial for young athletes. This approach supports sustained energy availability, preserves endogenous carbohydrate stores, and may optimize performance in extended physical activities, particularly for children in the early stages of maturation, but individual tolerance should guide its implementation.

For high-intensity activities and technical skill demands, sodium citrate may serve as a useful buffer against muscle acidosis in youth populations. A suggested dose of 0.3 to 0.5 g/kg, administered 60 to 90 min prior to exercise, supports sports requiring explosive power and precision, though individual tolerance should be monitored due to potential gastrointestinal discomfort.

Similarly, beetroot juice containing 5.4 mg/kg BM of nitrate demonstrated greater efficacy in recreationally active youth athletes. Consuming beetroot juice 2–3 h before exercise, or pairing it with other nutrients, may improve nitrate absorption and effectiveness, especially for activities demanding explosive strength. While its application appears promising, more research is needed to confirm its safety and efficacy in young athletes, and its use should be approached with caution.

## 7. Conclusions

This systematic review aimed to assess the acute effects of supplementation on performance in young athletes. The findings indicate that carbohydrate supplementation may enhance endurance performance in young athletes, likely by increasing glucose availability. Providing carbohydrate sources during exercise is particularly relevant for young athletes, as their energy reserves are smaller than those of adults due to their lower muscle volume. Additionally, these exogenous glucose sources may be essential for sparing muscle and liver glycogen, stabilizing blood glucose levels, and supplying energy to the brain, which also requires support during exertion. Sodium citrate supplementation improved technical performance. Beetroot juice demonstrated greater efficacy in recreationally active youth athletes. Variability in findings across studies may be attributed to methodological factors, such as dietary control and standardization, participant characteristics, protocols used, and measurement techniques. Young athletes, especially those in phases of growth and development, require tailored nutritional strategies that differ from adult guidelines due to distinct maturation stages and growth rates. These strategies should prioritize essential nutrients to support growth, development, and both physical and cognitive performance. This review seeks to clarify current knowledge on acute supplementation in young athletes, highlight existing evidence, identify research gaps, and inspire further studies to enhance youth sports performance.

## Figures and Tables

**Table 1:** Overview of the studies included in this systematic review.

Author	Country	Study Design	Sample	Substance/Placebo
Heidorn et al. [21]	USA	Randomized Placebo-Controlled	10 active girls (10 years old) Maturation: pubertal stage 1	Control: 19 g of CHO of sucrose and maltodextrin in the form of a 6% CHO drink. Just before and during exercise: 0.6 g/kg BM. PL: beverage sweetened with sucrose. Balanced meal (without control of carbohydrate intake) 3 or 4 h before.
Stough et al. [19]	Malaysia	Randomized, Crossover	58 healthy children boys and girls (between 10 and 12 years old) Maturation: not reported	11 g of malt extract, 6.5 g of milk, and 0.44 g of sucralose sweetener; 11 g of sucrose and 6.5 g of milk; 6.5 g of milk and 0.56 g of sucralose. Just before exercise: 0.3 g of CHO·kg^−1^ BM (approximate). PL: 150 mL of water and 0.56 g of sucralose. No control of food, suggesting that participants consume as usual.
Lopez-Samanes et al. [22]	Spain	Double-blind, Randomized, Placebo-controlled	10 competitive male basketball players (between 15 and 16 years old) Maturation: not reported	140 mL of beet juice with 400 mg nitrate. 3 h before: 5.2 mg/kg BM (approximate). PL: beet juice with low No3 concentration. All participants: Diet 60% CHO, 30% FAT, and 10% PTN.
Guth et al. [26]	USA	Double-blind Randomized, Placebo-controlled	8 active boys (11 years old) and 11 active men (23 years old) Maturation: pubertal stages 1 and 2	Control: 6% CHO solution (2.5 mg/kg BM–6 g of CHO and 100 mL of water). Just before exercise: 0.5 g/kg BM and during exercise: 0.3 g/kg BM (approximate). PL: colored water. All participants with the same controlled breakfast: Carnation^®^–12 g of CHO (60 kcal). No control of food, suggesting that participants consume as usual in the day before.
Cunha et al. [32]	Brazil	Double-blind, Crossover, Placebo-controlled	10 competitive male tennis players (17 years old) Maturation: not reported	Control: 0.5 g·kg^−1^ BM of sodium citrate. 2 h before exercise. PL: 0.1 g/kg BM of NaCl. All participants: Shake 2 g·kg^−1^ BM of CHO and 1 g·kg^−1^ BM of whey protein.
Bender et al. [27]	USA	Double-blind, crossover, Placebo-controlled	12 active male adolescents (16 years old) Maturation: not reported	2 × 70 mL of beet juice with 400 mg nitrate. 2.5 h before exercise: 5.4 mg/kg BM (approximate). PL: beet juice without nitrate. No control of food.
Russell et al. [23]	England	Double-blind, Crossover, Placebo-controlled	10 male soccer players (15 years old) Maturation: not reported	Control: 6% CHO solution (sucrose, sodium, and sodium chloride) + breakfast (54 g of CHO). 2 h before exercise: 1.3 g/kg BM (approximate). During exercise: 0.8 g/kg BM (approximate) in 8 occasions 14 mL/kg BM of 6% CHO solution. PL: flavored water (sodium and saccharin) + breakfast (54 g of CHO). Control of the diet in the two days before each test.
Phillips et al. [11]	England	Double-blind Randomized Counterbalanced	7 athletes of various sports (5 boys and 2 girls), 13 years old Maturation: pubertal stages 3 and 4	2% CHO-E solution (low CHO assay—LCHO); 6% CHO-E solution (moderate CHO assay—MCHO); 10% CHO-E solution (high CHO assay—HCHO); All solutions: sodium, 250 mg; magnesium, 60 mg; potassium, 90 mg; calcium, 20 mg. Just before exercise 5 mL/kg BM, during the effort 2 mL/kg BM each 15 min in the first hour. Just before and during exercise: 0.8 g/kg BM (approximate). Maintain the same diet the day before each test.
Phillips et al. [20]	England	Double-blind, Randomized Placebo-controlled	11 athletes of various sports (10 boys and 1 girl), 13 years old Maturation: pubertal stages 3 and 4	Control: 0.8 g/kg BM of CHO (maltodextrin) + 5 mL·kg^−1^ BM of water. Just before exercise. PL: artificially sweetened gel (without CHO) + 5 mL·kg^−1^ BM of water. Maintain the same diet the day before each test.
Carvalho et al. [25]	Portugal	Randomized, Crossover	12 male basketball players (16 years old) Maturation: not reported	A. No fluids (NF). B. Water intake ad libitum (W). C. Ad libitum intake of 8% carbohydrate-electrolytes (CSB), referring to an approximate value of 510 mL–41 g of CHO–0.5 g/kg BM during exercise. Maintain the same diet the day before each test.
Lee et al. [28]	USA	Double-blind, Crossover Placebo-controlled	13 boys athletes of various sports (15 years old) Maturation: pubertal stages > 3	Control: 1.5 g/kg BM of CHO (glucose) 30 min before exercise. PL: flavored water. All: breakfast 5 g FAT, 15 g CHO, and 12 g PTN (150 kcal).
Phillips et al. [24]	England	Double-blind, Randomized Placebo controlled	15 athletes of various sports (10 boys and 5 girls) Between 12 and 14 years old Maturation: pubertal stages 3 and 4	Control: 6% CHO-E solution (0.8 g/kg BM of maltodextrin) Just before exercise. PL: electrolytes only. All: 250 mg of sodium; 60 mg of magnesium; 90 mg of potassium and 20 mg of calcium. Maintain the same diet the day before each test.
Marjerrison et al. [29]	Canada	Randomized, Placebo-Controlled	11 boys (10 years old) Maturation: pubertal stages 1 and 2	Control: 1 g/kg BM of CHO (glucose) 30 min before exercise. PL: water. Light breakfast, maintain the same diet the day before each test.
Timmons and Bar-Or [30]	Canada	Double-blind, Counterbalanced, Placebo-Controlled	10 boys (10 years old) 10 men (20–25 years old) Maturation: pubertal stages 1 and 2	Control: 1.4 g/kg BM of 6% CHO solution (4% saccharose; 2% glucose) intermittently before and fractionated each 15 min during exercise. PL: flavored water. Boys: toast and jelly for breakfast (90 kcal)/Men: toast and jelly for breakfast (180 kcal).
Timmons et al. [13]	Canada	Double-blind, Randomized, Placebo-controlled	12 girls (12 years old) 10 teenagers (14 years old) Maturation: pubertal stages 1–4	Control: 1.7 g/kg BM of 6% CHO solution (4% saccharose; 2% glucose) subsequently consumed at 15-min intervals throughout exercise. PL: flavored water. All: toast and jelly for breakfast (90 kcal).
Timmons et al. [31]	Canada	Double-blind, Placebo-Controlled	20 boys (12 years old) 9 teenagers (14 years old) Maturation: pubertal stages 1–5	Control: 1.7 g/kg BM of 6% CHO solution (4% saccharose; 2% glucose) Just before and fractionated each 15 min during exercise. PL: flavored water. All: small breakfast with 12 mL/kg of beverage (0.7 g/kg BM of CHO).

**Table 2:** Overview of the assessment protocols and results in the included studies.

Author	Protocol and Performance Tests	Results
[21]	Before a 1-min performance sprint vs. treadmill exercise of varying intensity with two sets of 15 min, divided into 10 repeated sequences at 10%, 20%, 55%, and 95% of VO2max and a maximum 1-min sprint.	Compared to placebo, CHO supplementation did not alter physiological, perceptual, or performance responses during 30 min of variate-intensity treadmill exercise (VIE) or post-exercise sprint performance in pre-menarche girls.
[19]	Divided into 2–4 groups per session—30-min protocol. Total time spent on the 4 exercise tasks was 24 min with 90 s transition between each task. The four activities: badminton, squash, soccer, and “hopping relay” (playful). Attention and memory-image presentation, simple reaction time, choice reaction time, digit vigilance, numerical working memory, spatial working memory, and delayed image recognition.	The study demonstrated a beneficial effect of malt extract on attention accuracy and of sucrose on alertness, highlighting the importance of carbohydrates to alleviate attention changes due to exercise in children.
[22]	Counter movement jump (CMJ), isometric handgrip strength, a modified version of the T agility test, and 10 and 20 m sprint tests, followed by a monitored 40-min basketball game in which distances, speeds, accelerations, and decelerations were analyzed through GPS.	No significant differences were found between the results of consuming high-nitrate beet juice and low-nitrate placebo beet juice.
[26]	Applied a variable-intensity exercise protocol consisting of three 12-min cycles, with intensity varying every 20–30 s between 25%, 50%, 75%, and 125% of the VO2max work rate. Heart rate, perceived exertion, capillary blood glucose, lactate levels, and pulmonary gas exchange were monitored during the exercise.	The study concluded that, in this group of children, blood glucose levels and ratings of perceived exertion (RPE) were higher during the variable-intensity exercise (VIE) when carbohydrate (CHO) supplementation was used compared to placebo. However, blood lactate, heart rate, and pulmonary gas exchange were not significantly affected by the treatments.
[32]	The tests applied with this group of athletes were skill tests (tennis performance skill test—STPT, repeated sprint skill test—RSA) followed by a simulated 1-h match.	Compared to the placebo, the group supplemented with 0.5 g/kg of sodium citrate demonstrated greater shooting consistency, enhancing performance during a competition simulation.
[27]	Modified Wingate in four maximum effort sprints of 20 s against a resistance equivalent to 7.5% of the individual’s body mass, interspersed with 4 min of rest. Each participant was instructed to remain seated throughout the sprint. Data were collected at a sampling rate of 50 Hz on the Monark ATS software (Monark Ergomedic 894E, Vasbro, Sweden). Maximum power (PP) was defined as the highest power output obtained in watts (W), mean power output (MPO) was defined as the average power in W generated, and fatigue index (FI) was defined as the drop from PP to the lowest power during the sprint (W/s).	It was noticed that the administration of beetroot juice rich in nitrate can improve maximum power production in male adolescents.
[23]	Two soccer matches between the test team and a similar opposing team in the game pattern were organized for the purpose of this study, and each match lasted 90 min divided into two 45-min halves, which were separated by a 15-min passive recovery period.	Compared to the placebo, CHO (carbohydrate) supplementation increased blood glucose levels during the first half of exercise, accompanied by elevated lactate levels between 15 and 30 min. However, in the second half, blood glucose and lactate concentrations were comparable between the CHO and placebo conditions. Throughout the match, heart rate values and the percentage of time spent in different heart rate zones showed no significant differences between the CHO and placebo groups. These findings suggest that while CHO supplementation may have supported higher intensity during part of the first half, as indicated by elevated lactate levels, it did not result in an overall improvement in match intensity compared to the placebo.
[11]	The LIST was then completed, with participants performing four separate blocks separated by a 3 min recovery (part A), followed by an intermittent run to exhaustion (part B). The participants consumed the solution (2 mL kg^−1^ BM) in the recovery period between each 15-min block and in the recovery period before starting part B.	Time to exhaustion was significantly 34% higher with the 6% carbohydrate solution compared to the 10% solution. In the LIST results, mean sprint time and peak performance showed a tendency to improve with the 6% solution, although these differences did not reach statistical significance. The authors concluded that, in adolescents, the efficacy of carbohydrate supplementation does not appear to be fully optimized, consistent with findings observed in adults.
[20]	Two separate trials (divided into part A and part B). In the first trial, they completed four 15-min sessions (LIST), after which, an intermittent run to exhaustion (part B).	Gel ingestion did not significantly influence the average 15 m sprint time, peak sprint time, or heart rate. Despite this, the authors concluded that the ingestion of a CHO gel significantly increases intermittent endurance capacity during a simulated team game protocol.
[25]	Three separate 90 min training sessions. After each session, the athletes performed a set of basketball drills (2 points, 3 points, and free throws, suicide runs, and defensive zigzags).	Fluid restriction during exercise was associated with a higher level of dehydration and increased perception of effort but had no impact on basketball performance compared to ad libitum water intake or a CSB. However, after a 90-min training session, there were no differences in basketball performance among the no-water, ad libitum water, or 8% CHO-E solution intake conditions.
[28]	In two separate trials, subjects completed a Wingate Anaerobic Test (WAnT) protocol consisting of five 10-s sprints with 60 s of active recovery between each sprint.	The consumption of CHO prior to exercise was associated with improved medium peak power during the first Wingate Anaerobic Test (WAnT); however, this strategy did not mitigate the fatigue index when compared to the placebo.
[24]	Performance and capacity of high-intensity intermittent endurance of 15 collective sports athletes aged 12 years. In each test, they completed 60 min of exercise made up of four 15-min periods from (part A) of the LIST, divided as follows: 3× 20 m walking pace; 1 sprint; 3 × 20 m at 55% VO2max, and 3 × 20 m at 95% VO2max, followed by an intermittent run to exhaustion (part B).	Time to exhaustion was significantly 24% higher with the 6% CHO-E supplementation compared to the placebo. However, no differences in LIST performance were observed between the CHO-E and placebo conditions.
[29]	Four WAnTs at 2-min intervals on the cycle ergometer, with 5 s of unloaded pedaling at 60 rpm before the first WAnT. After that, each 2-min recovery period consisted of unloaded pedaling at 60 rpm.	Pre-exercise glucose levels were significantly higher in the CHO group compared to the placebo; however, no differences were observed in post-exercise glucose values. Additionally, the ingestion of a CHO solution prior to exercise did not influence power output or the fatigue index during repeated Wingate Anaerobic Test (WAnT) execution in pre- and early-pubertal boys.
[30]	On the bike at 70% VO2max in two series of 30 min with a 5-min interval between each.	CHO intake had no effect on VO_2_, VE, RR, or RPE in any of the groups. Heart rate was higher in the group that consumed CHO compared to the group that consumed water.
[13]	Aimed to determine substrate use during exercise with and without carbohydrate ingestion. The sample pedaled for 60 min at 70% of maximum aerobic power (VO2max).	Exogenous carbohydrate (CHOexo) intake was associated with lower post-exercise lactate levels in girls, suggesting reduced accumulation of metabolic by-products that could impair performance. CHOexo appears to be more effective in glycogen sparing, offering a direct benefit for prolonged and intense efforts in both groups.
[31]	In each trial, subjects cycled for 60 min at approximately 70% of their maximal aerobic power (VO2max). Substrate utilization during exercise was assessed in the final 15 min, using measurements of (13)C-enriched exogenous carbohydrate (CHO) consumption.	Prepubertal and early pubertal boys exhibited a higher reliance on exogenous carbohydrates (CHOexo), facilitating glycogen conservation. This adaptation may extend the capacity for sustained high-intensity exercise and postpone the onset of fatigue during prolonged physical activity. While mid- to late-pubertal boys showed higher endogenous carbohydrate (CHOendo) oxidation correlated with testosterone levels.

## Data Availability

The data that support the findings of this study are available from the corresponding author, NG.

## Supplementary Materials

The following supporting information can be downloaded at: https://www.mdpi.com/article/10.3390/nu16244304/s1, Table S1: Search Strategy and Filters.

## Abbreviations

BM: Body Mass; CHO: Carbohydrate; CHO-E: Carbohydrates with Electrolytes; CHO-endo: Endogenous Carbohydrate; CHO-exo: Exogenous Carbohydrate; HR: Heart Rate; PL: Placebo; PRISMA: Preferred Reporting Items for Systematic Reviews and Meta-Analyses; RPE: Rating of Perceived Exertion; RR: Respiratory Rate VE: Minute Ventilation; VIE: Variable Intensity Exercise; WAnT: Wingate Test.
