# Supplementary material for: Enhancing Performance in Young Athletes: A Systematic Review of Acute Supplementation Effects
Source: Nutrients. 2024 Dec 13;16(24):4304. doi: 10.3390/nu16244304 (PMC11679880; doi:10.3390/nu16244304)
Supplement: Supplementary file 1 [file nutrients-16-04304-s001.zip › nutrients-3293032-supplementary.pdf]

Database	Search Strategy and Filters	Articles Identified
Web of Science	Performance (Abstract) AND “ergogenic effect” OR “ergogenic aid” OR “ergogenic substance$” OR “dietary supplement$” OR “food supplement*” OR carbo* OR resveratrol OR taurine OR beetroot OR ATP OR phosphocreatine OR choline OR magnesium OR vitamin* (Abstract) AND Sport* OR exercis* OR athletic$ OR soccer OR swim* OR tennis OR gymnastic* OR judo OR basketball OR rugby OR football OR “team sport” (Abstract) AND youth OR young OR kid* OR Child* OR "pre puberty" OR "young athlete" (Abstract)	468
SCOPUS	(TITLE-ABS-KEY (sport* OR exercis* OR athletic$ OR soccer OR swim* OR tennis OR gymnastic* OR judo OR basketball OR rugby OR football OR "team sport") AND ABS ("ergogenic effect" OR "ergogenic aid" OR "ergogenic substance$" OR "dietary supplement$" OR "food supplement*" OR carbo* OR resveratrol OR taurine OR beetroot OR ATP OR phosphocreatine OR choline OR magnesium OR vitamin*) AND TITLE-ABS-KEY (performance) AND TITLE-ABS-KEY (youth OR young OR kid* OR child* OR "pre puberty" OR "young athlete"))	1822
PubMed	(((Sport* OR exercis* OR athletic$ OR soccer OR swim* OR tennis OR gymnastic* OR judo OR basketball OR rugby OR football OR "team sport") AND ("ergogenic effect" OR "ergogenic aid" OR "ergogenic substance$" OR "dietary supplement$" OR "food supplement*" OR carbo* OR resveratrol OR taurine OR beetroot OR ATP OR phosphocreatine OR choline OR magnesium OR vitamin*)) AND (Performance)) AND (youth OR young OR kid* OR Child* OR "pre puberty" OR "young athlete"))	256

Supplementary Table 1. Search Strategy and Results
